# Clinical Performance of Lithium Disilicate Ceramic Veneers Cemented With Light-Cured Resin Cements: An Observational Study

**DOI:** 10.7759/cureus.83862

**Published:** 2025-05-10

**Authors:** Nguyen Thi Minh Hien, Tran Hung Lam, Do Thi Thao, Hoang Viet

**Affiliations:** 1 Faculty of Dentistry, Can Tho University of Medicine and Pharmacy, Can Tho, VNM; 2 Department of Prosthodontics, Faculty of Dentistry, Van Lang University, Ho Chi Minh City, VNM; 3 Department of Oral Pathology and Periodontology, Faculty of Dentistry, Can Tho University of Medicine and Pharmacy, Can Tho, VNM; 4 Department of Orthodontics and Pedodontics, Faculty of Dentistry, Van Lang University, Ho Chi Minh City, VNM

**Keywords:** ceramic veneers, clinical performance, lithium disilicate, marginal adaptation, resin cement

## Abstract

Objective: This study aimed to evaluate and compare the clinical performance of ceramic veneers cemented with two different cements, Variolink Esthetic LC (Ivoclar, Schaan, Liechtenstein) and Vitique (DMG, Hamburg, Germany), over a three-month period.

Methods: Sixty patients (49 females and 11 males; age range = 18-45 years; mean age = 34.93 ± 7.48 years) requiring ceramic veneer restorations on anterior teeth were divided into two groups based on the cement used: Variolink (n = 30) and Vitique (n = 30). All the veneers were fabricated using lithium disilicate with a thickness between 0.5 and 0.7 mm. Clinical evaluations were performed at one week (T1) and three months (T2) after placement using the modified plaque index (mPLI), modified bleeding index (mBI), color match between restored and adjacent teeth using spectrophotometry (VITA Easyshade V, VITA Zahnfabrik, Bad Säckingen, Germany), and the modified United States Public Health Service (USPHS) criteria.

Results: At T1, 91.7% (n = 55) of cases showed no plaque (score 0), which improved to 96.7% (n = 58) at T2. Regarding the bleeding index, 95.0% of patients (n = 57) showed no bleeding (score 0) at T1, which improved to 98.3% (n = 59) at T2. The color difference (ΔE) between the restored and adjacent teeth at T1 was 2.13 ± 2.17 for Variolink and 1.91 ± 1.79 for Vitique (p = 0.673), with similar values at T2 (2.30 ± 1.42 and 2.22 ± 1.54, respectively; p = 0.751). Central incisors showed significantly better color match (ΔE = 2.10 ± 1.92) than lateral incisors (ΔE = 2.62 ± 2.08) at T2 (p = 0.04). Regarding marginal adaptation, most restorations showed perfect adaptation or minor discrepancies. Marginal discoloration was minimal.

Conclusion: This study demonstrated the excellent short-term performance of Variolink and Vitique. Both cement systems provided good marginal adaptation, color stability, and biocompatibility, with no significant differences between them.

## Introduction

Aesthetic dentistry merges art and science to enhance oral health and function, with a primary focus on achieving significant aesthetic improvements while minimizing invasive procedures [1]. Among the various restorative options, ceramic veneers have gained popularity for anterior teeth because of their natural appearance, excellent aesthetic qualities, and conservative preparation requirements compared with full-coverage crowns [2,3]. These thin ceramic shells effectively address a range of dental issues, including diastema closure, correction of minor misalignments, restoration of fractured teeth, and masking of discoloration. Notably, ceramic veneers have demonstrated survival rates exceeding 90% at 10 years in well-selected cases, highlighting their reliability in aesthetic dentistry [4].

The clinical success of ceramic veneers depends on multiple factors, including proper case selection, meticulous preparation design, appropriate material selection, precise laboratory fabrication, and, perhaps most critically, the cementation protocol [5,6]. The choice of cement significantly influences both the immediate aesthetic outcome and the long-term durability of the restoration. Lithium disilicate has emerged as a leading material for ceramic veneers because of its superior mechanical properties, including a flexural strength of 350-400 MPa, and excellent optical characteristics that closely mimic natural dentition [7,8]. Additionally, its ability to be fabricated in thin sections (0.5-0.7 mm) and reliable bonding to tooth structures through established adhesive protocols make it an ideal choice.

Cementation is a critical step in ensuring the success of ceramic veneers [9]. Contemporary resin cements, such as Variolink Esthetic LC (Ivoclar, Schaan, Liechtenstein) and Vitique (DMG, Hamburg, Germany), offer advantages such as high bond strength to both tooth structure and ceramic surfaces, improved color stability, and simplified application protocols. Despite these benefits, there is a notable lack of comparative clinical studies evaluating different cement systems under standardized conditions, which creates a gap in our understanding of their relative performance in real-world clinical scenarios [10]. Therefore, this study was designed to address the existing knowledge gap by prospectively evaluating and comparing the short-term clinical performance of lithium disilicate ceramic veneers cemented with two contemporary light-cured resin cements: Variolink Esthetic LC and Vitique. To achieve this, a comprehensive set of clinical parameters was assessed over a three-month follow-up period, including periodontal health indicators (modified plaque index and modified bleeding index), color matching and stability (measured by ΔE values using spectrophotometry), marginal adaptation and discoloration (using the modified United States Public Health Service criteria), and the presence of biological and mechanical complications such as postoperative sensitivity, secondary caries, and restoration fractures. Through this approach, the study aimed to provide clinically relevant evidence regarding the effectiveness, biocompatibility, and aesthetic outcomes associated with each cement system, thereby offering guidance for clinicians in selecting optimal luting agents for anterior ceramic veneer restorations.

## Materials and methods

Study design

This observational study was conducted from September 2023 to June 2024 at the Department of Odonto-Stomatology, Van Lang University, and EMCAS Hospital in Ho Chi Minh City. The study received ethical approval from the institutional review boards under decision numbers 1465/QD-DHYDCT and 23.399.HV/PCT-HDDD.

Sample size

The sample size was estimated using the formula for estimating one mean:

$n = \frac{{Z_{1 - \alpha/2}^{2} \cdot \sigma^{2}}}{{d^{2}}}$

Where *n* is the sample size and *Z* is the standard distribution value, calculated based on the significance level α. For *α* = 0.05, *Z* = 1.96; *σ* is the standard deviation of ΔE. According to a study by Dadashi et al. [11], *σ* = 0.9 and *d* = 0.341. Thus, the minimum sample size required for the study was 60 teeth that required porcelain veneer restorations (30 for each group).

Inclusion and exclusion criteria

The study involved participants between the ages of 18 and 45 years who required dental tissue restoration using porcelain veneers on one of the four upper permanent incisors. Eligible participants needed to be free from periodontal diseases, such as severe gingivitis or periodontitis, and have vital pulp. On the other hand, individuals were excluded from the study if they had habits that applied excessive force on restorations, such as uncontrolled bruxism, tooth clenching, or nail biting. Additional exclusion criteria included severe tooth wear, insufficient supporting enamel, previous endodontic treatment, significant tooth fractures with inadequate hard tissue, severe surface staining (including discolored enamel, fluorosis, or tetracycline staining), poor oral hygiene, and severely displaced dental anatomy with moderate to severe tooth misalignment.

In our country, aesthetic restorations utilizing ceramic veneers are not considered a medical procedure to treat a disease. Therefore, they are not covered by insurance, and patients must pay the cost themselves. At our dental clinic, esthetic restorations utilizing ceramic veneers are provided as elective procedures based on patient demand. Following a clinical consultation, patients selected the luting material to be used: either Variolink Esthetic LC or Vitique. The dentist did not prescribe or recommend any specific type of ceramic veneers to the patients.

This study included 60 patients (n = 30 per group; 49 females and 11 males) between 18 and 45 years of age, all of whom required esthetic restoration of the maxillary anterior teeth with ceramic veneers.

All the porcelain veneers in this study were fabricated from lithium disilicate (IPS e.max Press, Ivoclar) with a uniform thickness ranging from 0.5 to 0.7 mm. Clinical evaluations were conducted at two time points: one week (T1) and three months (T2) after cementation. Color values were measured using a spectrophotometer (VITA Easyshade V, VITA Zahnfabrik, Bad Säckingen, Germany) utilizing the CIELAB color system with three parameters: a∗, b∗, and L∗. Where L∗ is the vertical axis representing lightness with values ranging from 0 (black) to +100 (white). a∗ and b∗ are two axes lying on a plane perpendicular to the L∗ axis, containing the four basic colors visible to the human eye: green (-a∗), red (+a∗), blue (-b∗), and yellow (+b∗). Each a∗ and b∗ has values ranging from -128 to 128.

The color difference between the measurement stages (T1 and T2) was expressed through the delta E (ΔE) value, which was measured according to the International Commission on Illumination (CIE) 1976 formula [12].

∆E = [(∆L*)2 + (∆a*)2 + (∆b*)2]1⁄2

Assessment parameters included the modified plaque index (mPLI) [13]. The mPLI scores ranged from 0 (no plaque) to 3 (abundant plaque and food debris), whereas the modified bleeding index (mBI) ranged from 0 (no bleeding) to 3 (profuse bleeding). Color matching between the restored and adjacent teeth was objectively measured using a spectrophotometer (VITA Easyshade V). Restorations were evaluated using the modified United States Public Health Service (USPHS) criteria [14], which included assessments of marginal adaptation, marginal discoloration, restoration and tooth fractures, secondary caries, and postoperative sensitivity.

Data statistics

All collected data were organized in Microsoft Excel (Microsoft Corp., Redmond, WA) and subsequently analyzed using IBM SPSS Statistics version 26 (IBM Corp., Armonk, NY). To assess differences within each group over time, a paired sample t-test was applied. For comparisons between two independent groups, either the independent samples t-test or the Mann-Whitney U test was used, depending on the data distribution. Statistical significance was set at p < 0.05.

## Results

Plaque and bleeding indices

At T1, 91.7% (n = 55) of the cases showed no plaque (score 0), and 8.3% (n = 5) showed minimal plaque (score 1). At T2, these values had improved to 96.7% (n = 58) and 3.3% (n = 2), respectively. None of the cases showed visible plaques (scores 2-3) at either time point. Regarding the bleeding index, 95.0% (n = 57) of the cases showed no bleeding (score 0), and 5.0% (n = 3) showed isolated bleeding points (score 1) at T1. At T2, these values had improved to 98.3% (n = 59) and 1.7% (n = 1), respectively. None of the cases showed confluent or profuse bleeding (scores 2-3) at either time point (Table 1).

**Table 1:** Plaque and bleeding indices according to age group, sex, and tooth position of restoration. Time point: T1: after one week; T2: after three months.

Variable		Time point	Plaque index		Bleeding index	
			No plaque, n (%)	Minimal plaque, n (%)	No bleeding, n (%)	Localized bleeding, n (%)
		T1 (n = 60)	55 (91.7)	5 (8.3)	57 (95.0)	3 (5.0)
		T2 (n = 60)	58 (96.7)	2 (3.3)	59 (98.3)	1 (1.7)
Age group	15-<30	T1 (n = 16)	14 (87.5)	2 (12.5)	13 (81.2)	3 (18.8)
		T2 (n = 16)	16 (100.0)	0 (0.0)	15 (93.8)	1 (6.2)
	30-45	T1 (n = 44)	41 (93.2)	3 (6.8)	44 (100.0)	0 (0.0)
		T2 (n = 44)	42 (95.5)	2 (4.5)	44 (100.0)	0 (0.0)
Sex	Male	T1 (n = 11)	8 (72.7)	3 (27.3)	10 (90.9)	1 (9.1)
		T2 (n = 11)	9 (81.8)	2 (18.2)	10 (90.9)	1 (9.1)
	Female	T1 (n = 49)	47 (95.9)	2 (4.1)	47 (95.9)	2 (4.1)
		T2 (n = 49)	49 (100.0)	0 (0.0)	49 (100.0)	0 (0.0)
Tooth position of restoration	Central incisor	T1 (n = 47)	45 (95.7)	2 (4.3)	44 (93.6)	3 (6.4)
		T2 (n = 47)	46 (97.9)	1 (2.1)	46 (97.9)	1 (2.1)
	Lateral incisor	T1 (n = 13)	10 (76.9)	3 (23.1)	13 (100.0)	0 (0.0)
		T2 (n = 13)	12 (92.3)	1 (7.7)	13 (100.0)	0 (0.0)

Color match with adjacent teeth

The color difference (ΔE) between restored and adjacent teeth at T1 was 2.13 ± 2.17 for Variolink and 1.91 ± 1.79 for Vitique (p = 0.673). At T2, these values were 2.30 ± 1.42 and 2.22 ± 1.54, respectively (p = 0.751), indicating no significant difference between the two cement systems (Table 2). When analyzed by tooth position, central incisors showed better color match (ΔE = 2.10 ± 1.92) than lateral incisors (ΔE = 2.62 ± 2.08) at T2, with a statistically significant difference (p = 0.04) (Table 3).

**Table 2:** Color compatibility (∆E) between restorations and adjacent teeth. * Independent t-test; ** paired t-test. Time point: T1: after one week; T2: after three months.

Time point	Type of cement, mean ± SD		p*	t-value
	Variolink	Vitique		
T1	2.13 ± 2.17	1.91 ± 1.79	0.673	0.4
T2	2.30 ± 1.42	2.22 ± 1.54	0.751	0.33
p**	0.326	0.409	-	-
t-value	0.98	0.83	-	-

**Table 3:** Color compatibility (∆E) between restorations and adjacent teeth age group, sex, and tooth position of restoration. Time point: T1: after one week; T2: after three months. ∆E is expressed as mean ± SD. P-value was obtained using the independent t-test.

Cement type	Time point	Age				Sex				Tooth position of restoration			
		15-<30 (n = 16)	30-45 (n = 44)	p	t-value	Male (n = 11)	Female (n = 49)	p	t-value	Central incisor (n = 47)	Lateral incisor (n = 13)	p	t-value
Variolink	T1	1.95 ± 1.19	2.19 ± 1.16	0.3	1.05	2.33 ± 1.26	2.09 ± 1.15	0.4	0.9	1.99 ± 1.67	2.32 ± 1.82	0.3	1.04
	T2	2.42 ± 1.35	2.32 ± 1.19	0.4	0.8	2.35 ± 1.24	2.34 ± 1.22	0.7	0.4	2.13 ± 1.74	2.64 ± 1.96	0.05	2.0
Vitique	T1	1.46 ± 0.95	2.1 ± 1.26	0.1	1.7	2.02 ± 1.27	1.87 ± 0.5	0.6	0.5	1.86 ± 1.45	2.33 ± 1.84	0.2	1.3
	T2	1.84 ± 1.19	2.16 ± 1.25	0.2	1.2	2.11 ± 1.27	1.81 ± 1.06	0.3	1.03	2.04 ± 1.64	2.61 ± 1.81	0.04	2.1

Marginal adaptation and discoloration

At both T1 and T2, 91.7% (n = 55) of the restorations showed perfect marginal adaptation (score 0), and 8.3% (n = 5) showed minor discrepancies (score 1). None of the cases showed more severe marginal defects (scores 2-4). For marginal discoloration, at T1, 91.7% (n = 55) showed no discoloration (score 0) and 8.3% (n = 5) showed slight staining that could be polished away (score 1). At T2, 100% (n = 60) of patients showed no discoloration (Table 4).

**Table 4:** Marginal adaptation and discoloration. Time point: T1: after one week; T2: after three months.

Variable	Point	Time point, n (%)	
		T1	T2
Marginal discoloration	0	55 (91.7)	60 (100.0)
	1	5 (8.3)	0 (0.0)
Marginal adaptation	0	55 (91.7)	55 (91.7)
	1	5 (8.3)	5 (8.3)

Restoration, tooth fracture, secondary caries, and sensitivity

No cases of restoration or tooth fracture were observed at T1 or T2. No cases of secondary caries were observed at any time point. At T1, 5.0% of the cases showed mild sensitivity (score 1), which resolved completely by T2 (0%).

## Discussion

This prospective clinical study demonstrated the excellent short-term performance of lithium disilicate ceramic veneers cemented with either Variolink Esthetic LC or Vitique. Both resin cement systems showed comparable clinical outcomes across key parameters, including periodontal health, color match, marginal adaptation, and mechanical stability.

Improvement in plaque and bleeding indices from T1 to T2 suggests the biocompatibility of both cements and successful patient adaptation to the restorations. Specifically, plaque accumulation decreased from 8.3% to 3.3%, and bleeding on probing dropped from 5.0% to 1.7% within three months. This favorable periodontal response can be attributed to multiple factors. The smooth surface of lithium disilicate ceramics, precise marginal adaptation, reduced plaque retention niches, and improved patient oral hygiene motivation all played important roles. Additionally, careful finishing and polishing of restoration margins during cementation likely enhanced soft tissue compatibility, as supported by previous studies demonstrating the significance of marginal smoothness for periodontal health [10,15].

To control potential confounding factors, a standardized oral hygiene protocol was implemented for all participants. Patients received professional scaling, detailed instructions before and after veneer placement, and regular reinforcement during follow-up visits. This approach ensured consistent hygiene levels across the cohort, allowing observed differences to be attributed primarily to material and restoration factors rather than individual variations in home care.

While surface characteristics and hygiene are critical, resin cement properties also influence periodontal health outcomes. Despite meticulous removal of excess cement, the inherent features of the resin cements, such as surface smoothness, polymerization shrinkage behavior, and marginal sealing ability, likely contributed to the favorable clinical results. Light-cured resin cements like Variolink and Vitique, with optimized filler content and advanced polymerization mechanisms, provide stable, smooth, and biocompatible marginal interfaces. These attributes reduce bacterial adhesion, enhance gingival health, and explain the positive mPLI and mBI scores observed in this study.

The margin location also played an important role. All veneers were designed with supragingival or equigingival margins to avoid subgingival trauma, facilitate patient hygiene, and reduce the risk of plaque-induced inflammation. This design not only supports soft tissue health but also enables reliable clinical evaluation of marginal adaptation and discoloration. Collectively, margin positioning, favorable cement properties, and careful clinical protocols synergistically contributed to the optimal periodontal outcomes achieved.

Color match and stability were equally favorable. Both cement systems demonstrated clinically acceptable color integration, with ΔE values well below the perceptibility threshold (3.3 units) at both T1 and T2. The slight increase in ΔE over time remained insignificant, indicating excellent short-term color stability. These findings align with previous studies suggesting superior color retention of light-cured resin cements over dual-cured variants due to the absence of aromatic amines and inclusion of UV stabilizers [16,17]. Interestingly, color match was slightly better for central incisors than lateral incisors at three months. This likely reflects the greater optical complexity of lateral incisors, including thinner enamel and more variable dentin shades [18,19]. Such findings emphasize the need for clinicians to consider tooth-specific optical properties when selecting veneer and cement combinations, and highlight the value of digital shade matching techniques in challenging cases.

Marginal adaptation was excellent throughout the observation period. At both one week and three months, 91.7% of restorations demonstrated perfect marginal adaptation, and none required intervention. This suggests that modern fabrication techniques (e.g., computer-aided design/computer-aided manufacturing and pressing), combined with favorable cement handling and polymerization characteristics, provided durable and accurate marginal seals [7,20]. Furthermore, marginal discoloration initially noted in some cases resolved completely by three months following routine polishing, further confirming the restorations’ integrity and stability.

In terms of mechanical reliability, no restoration or tooth fractures were observed during the study. This supports the well-documented strength of lithium disilicate ceramics, even at reduced veneer thicknesses (0.5-0.7 mm) [3,4]. Combined with conservative preparation designs that preserved enamel and excluded high-risk patients (e.g., bruxism and severe malocclusion), the use of these ceramics and cements offers a predictable approach for anterior aesthetic restorations with minimal risk of early mechanical failure.

While no secondary caries were detected during the short-term follow-up, this parameter was nevertheless assessed in accordance with the USPHS criteria. Although carious lesions typically develop over longer periods, early signs of marginal failure, such as staining or softening, can provide important prognostic indicators. The absence of such signs during this study reinforces the high-quality marginal seals achieved and underscores the excellent short-term biological performance of both resin cement systems [21].

The study had several limitations. The relatively short follow-up period of three months restricts the ability to draw conclusions regarding long-term clinical outcomes, including potential complications such as secondary caries, restoration fractures, and color instability. Moreover, the study involved a specific patient population with optimal hygiene, supragingival or equigingival margins, and minimal tooth discoloration, limiting the generalizability of results to more complex clinical scenarios. Additionally, while the sample size was sufficient to detect major differences between groups, larger-scale studies are needed to evaluate subtle performance variations and long-term durability. Future research should aim to address these limitations by investigating longer follow-up periods, a wider range of ceramic and cement combinations, and more diverse patient conditions, including subgingival margins and high-risk periodontal environments.

## Conclusions

This prospective clinical study demonstrated that lithium disilicate ceramic veneers cemented with either Variolink Esthetic LC or Vitique exhibited excellent short-term clinical performance. Both resin cements provided comparable outcomes in terms of marginal adaptation, color stability, and biocompatibility. In particular, favorable periodontal health was observed, with minimal plaque accumulation, low bleeding indices, and an absence of gingival inflammation throughout the three-month follow-up period. These results highlight the importance of resin cement properties, alongside ceramic surface smoothness and margin design, in supporting soft tissue health in aesthetic restorations.

Nevertheless, these findings should be interpreted with caution given the short-term nature of the study. While no biological or mechanical complications, such as secondary caries or restoration fractures, were observed, such events often occur over longer timeframes. Thus, the favorable clinical outcomes reported here primarily reflect early success and do not fully predict long-term performance. Furthermore, the study was conducted under standardized oral hygiene protocols and involved veneers placed at supragingival or equigingival margins, which may not reflect more challenging clinical conditions. Future studies with extended observation periods are essential to assess the durability of periodontal health, color stability, and marginal integrity over time. In addition, research involving more diverse clinical situations, including subgingival margins or higher-risk patients, would provide deeper insights into the long-term clinical behavior of different resin cement systems. Despite these limitations, the present study offers valuable preliminary evidence supporting the short-term reliability and biological compatibility of modern light-cured resin cements in anterior aesthetic veneer restorations.
